# Supplementary figures and images for: Using embedded alginate microparticles to tune the properties of in situ forming poly(N‐isopropylacrylamide)‐graft‐chondroitin sulfate bioadhesive hydrogels for replacement and repair of the nucleus pulposus of the intervertebral disc
Source: JOR Spine. 2021 Jun 1;4(3):e1161. doi: 10.1002/jsp2.1161 (PMC8479524; doi:10.1002/jsp2.1161)

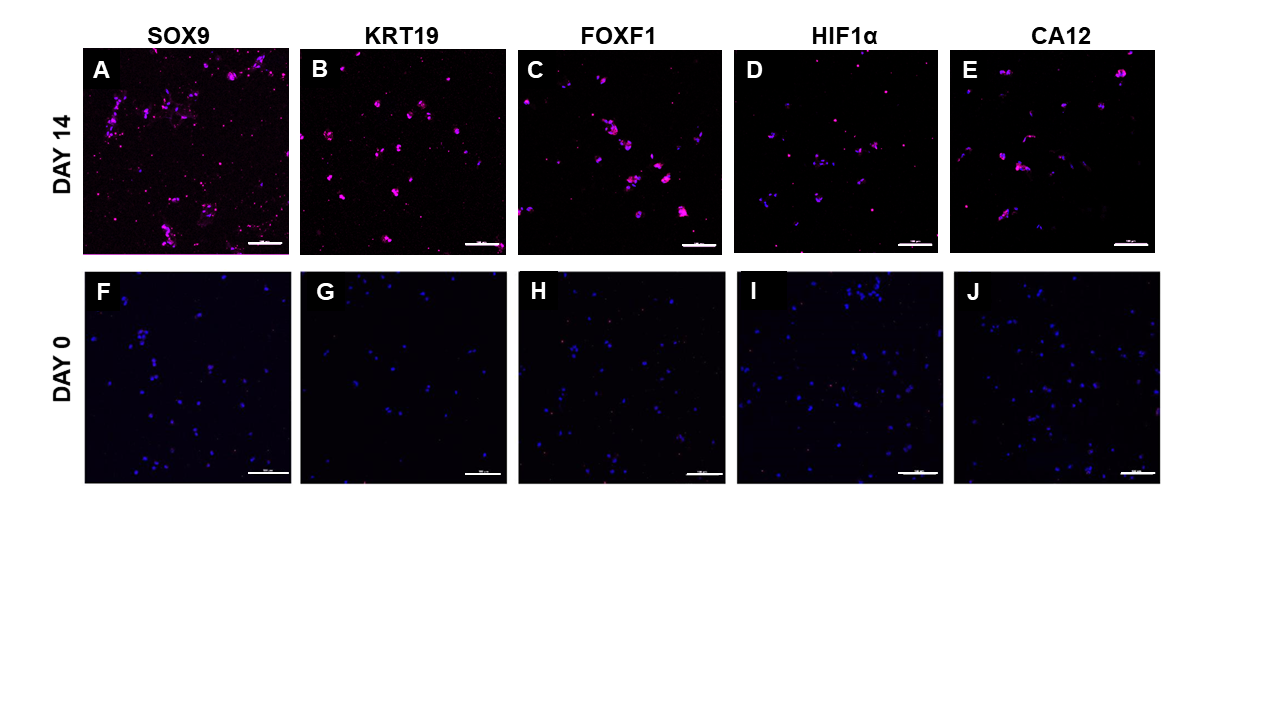

Supplement: Supplementary file 1 — Figure S1 Representative immunofluorescent staining (magenta) of (A) SOX9, (B) KRT19, (C) FOXF1, (D) HIF1α, and (E) CA12 produced by ADMSCs cultured within S‐50 for 14 days in the presence of soluble GDF‐6. Staining for day 0, immediately after encapsulation within the bioadhesive, is presented as a comparison in (F‐J). Cell nuclei are counterstained with DAPI (blue). Scale bars = 100 μm. [file JSP2-4-e1161-s002.png]

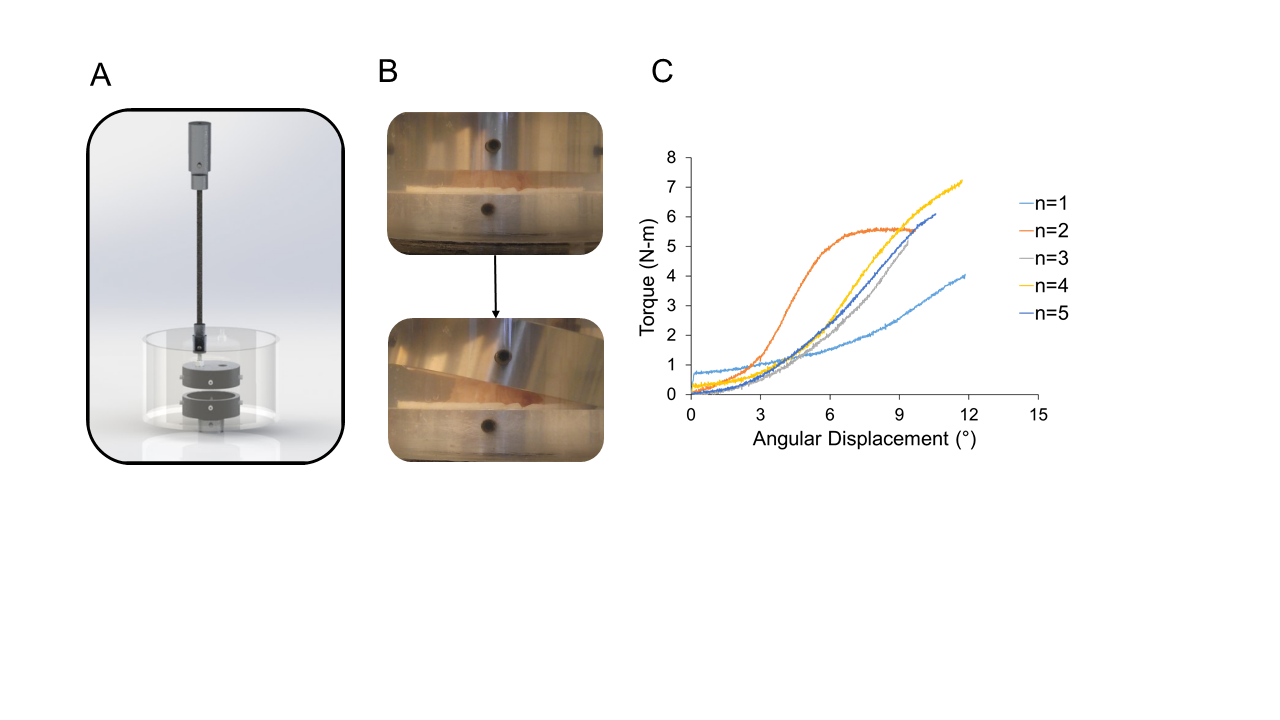

Supplement: Supplementary file 2 — Figure S2(A) Custom‐made mechanical fixtures designed to induce lateral bending of the IVD specimen. The vertical rod is offset 25.4 mm from the center of the stainless steel cup and affixed to a freely‐rotating hinge allowing for rotational movement. (B) High magnitude extrusion test where the angle was continuously increased at a rate of 0.1°/sec on the side opposite to the injection site. The test was stopped manually when the maximum bending angle was reached due to geometric constraints of the tissue. (C) Torque vs angular displacement curves for n = 5 repeats of the high magnitude extrusion test. The specimens were compressed to average maximum angle of 11.2 ± 1.2° with no evidence of herniation. [file JSP2-4-e1161-s001.png]
