# Supplementary material for: Using embedded alginate microparticles to tune the properties of in situ forming poly(N‐isopropylacrylamide)‐graft‐chondroitin sulfate bioadhesive hydrogels for replacement and repair of the nucleus pulposus of the intervertebral disc
Source: JOR Spine. 2021 Jun 1;4(3):e1161. doi: 10.1002/jsp2.1161 (PMC8479524; doi:10.1002/jsp2.1161)
Supplement: Supplementary file 3 — Table S1 Genes of interest for ADMSCs cultured within formulation S‐50 for 14 days in the presence of soluble GDF‐6. Table S2. Proteins of interest for immunofluorescent labeling of ADMSCs cultured within formulation S‐50 for 14 days in the presence of soluble GDF‐6. [file JSP2-4-e1161-s003.docx]

**Supplementary Table 1.** Genes of interest for ADMSCs cultured within formulation S-50 for 14 days in the presence of soluble GDF-6.

| **Gene** | **Forward Primer (5’ – 3’)** | **Reverse Primer (5’ – 3’)** | **Product Size (bp)** |
| --- | --- | --- | --- |
| COL1 | CCTGCTGGCAAGAGTGGTGAT | GAAGCCACGGTGACCCTTTATG | 165 |
| COL2 | GGCAATAGCAGGTTCACGTACA | CGATAACAGTCTTGCCCCACTT | 79 |
| ACAN | TCGAGGACAGCGAGGCC | TCGAGGGTGTAGCGTGTAGAGA | 85 |
| SOX9 | AGCGAACGCACATCAAGAC | CTGTAGGCGATCTGTTGGGG | 85 |
| KRT19 | GATAGTGAGCGGCAGAATCA | CCTCCAAAGGACAGCAGAAG | 178 |
| CA12 | CGTGCTCCTGCTGGTGATCT | AGTCCACTTGGAACCGTTCACT | 70 |
| HIF1α | GGGTTGAAACTCAAGCAACTGTC | GTGCTGAATAATACCACTCACAACG | 98 |
| FOXF1 | AAGCCGCCCTATTCCTACATC | GCGCTTGGTGGGTGAACT | 63 |
| PAX1 | TGGCCCTCGGCACACTC | GCCCCTGTTTGCTCCATAAA | 65 |
| GAPDH | CAGCGACACCCACTCCTC | TGAGGTCCACCACCCTGT | 122 |

**Supplementary Table 2**. Proteins of interest for immunofluorescent labeling of ADMSCs cultured within formulation S-50 for 14 days in the presence of soluble GDF-6.

| **Protein** | **Manufacturer** | **Antibody Type** | **Species** | **Clonality** | **Dilution** |
| --- | --- | --- | --- | --- | --- |
| COL1 | ab90395 | Primary | Mouse anti-human | Monoclonal | 1:100 |
| COL2 | ab185430 | Primary | Mouse anti-human | Monoclonal | 1:200 |
| ACAN | ab3778 | Primary | Mouse anti-human | Monoclonal | 1:50 |
| SOX9 | ab76997 | Primary | Mouse anti-human | Monoclonal | 1:100 |
| KRT19 | ab7754 | Primary | Mouse anti-human | Monoclonal | 1:200 |
| CA12 | ab195233 | Primary | Rabbit anti-human | Monoclonal | 1:50 |
| HIF1α | ab51608 | Primary | Rabbit anti-human | Monoclonal | 1:100 |
| FOXF1 | ab168383 | Primary | Rabbit anti-human | Monoclonal | 1:100 |
